# Clinical and genetic characterization of pediatric patients with Wilson’s disease from Yunnan province where ethnic minorities gather

**DOI:** 10.3389/fgene.2023.1142968

**Published:** 2023-03-20

**Authors:** Yanjun Wang, Jiahui Fang, Bin Li, Chongyang Li, Shan Liu, Juan He, Lvyan Tao, Cuifen Li, Ya Yang, Li Li, Shufang Xiao

**Affiliations:** ^1^ Kunming Children’s Hospital, Kunming, China; ^2^ Department of Oncology, The Affiliated Hospital of Yunnan University, Kunming, China; ^3^ Yunnan Cancer Hospital, Kunming, China

**Keywords:** Wilson’s disease, pediatric patients, ATP7B, genetic characterization, ethnic minorities

## Abstract

**Background:** Wilson’s disease (WD) is an autosomal recessive disease that is caused by mutations in the *ATP7B* (a copper-transporting P-type ATPase) gene. The disease has a low prevalence and is characterized by a copper metabolism disorder. However, various characteristics of the disease are determined by race and geographic region. We aimed to discover novel *ATP7B* mutations in pediatric patients with WD from Yunnan province, where there is a high proportion of ethnic minorities. We also performed a comprehensive analysis of *ATP7B* mutations in the different ethnic groups found in Southwest China.

**Methods:** We recruited 45 patients who had been clinically diagnosed with WD, from 44 unrelated families. Routine clinical examinations and laboratory evaluations were performed and details of age, gender, ethnic group and symptoms at onset were collected. Direct sequencing of the *ATP7B* gene was performed in 39 of the 45 patients and their families.

**Results**: In this study, participants came from seven different ethnic groups in China: Han, Bai, Dai, Zhuang, Yi, Hui and Jingpo. Three out of ten patients from ethnic minorities presented with elevated transaminases, when compared to the majority of the Han patients. Forty distinct mutations (28 missense, six splicing, three non-sense, two frameshift and one mutation of uncertain significance) were identified in the 39 patients with WD. Four of the mutations were novel and the most frequent mutation was c.2333G > T (p.R778L, allelic frequency: 15.38%). Using the phenotype-genotype correlation analysis, patients from ethnic minorities were shown to be more likely to have homozygous mutations (*p* = 0.035) than Han patients. The patients who carried the c.2310C > G mutation had lower serum ceruloplasmin levels (*p* = 0.012). In patients with heterozygous mutations, c.3809A > G was significantly associated with ethnic minorities (*p* = 0.042). The frequency of a protein-truncating variant (PTV) in Han patients was 34.38% (11/32), while we did not find PTV in patients from ethnic minorities.

**Conclusion**: This study revealed genetic defects in 39 pediatric patients with WD from Yunnan province. Four novel mutations were identified and have enriched the WD database. We characterized the genotypes and phenotypes in different minorities, which will enhance the current knowledge on the population genetics of WD in China.

## Introduction

Wilson’s disease (WD; OMIM 277900) is an inherited, autosomal recessive copper metabolism disorder that is caused by mutations in the *ATP7B* gene (Cai et al., 2022). The age of onset ranges from eightmonths to 74 years (Ohura et al., 1999; Hahn et al., 2002; Ala et al., 2007; Abuduxikuer et al., 2015). The worldwide prevalence of this disease has been estimated to be approximately 1/100,000 to 3/100,000, whilst it is more prevalent in East Asia, where it is estimated to occur at a prevalence of 1/1,500 to 1/3,000 (Sandahl et al., 2020; Wallace and Dooley, 2020; Li et al., 2022). The carrier frequency of WD is 1/90 and heterozygous mutations are found in up to 2.5% of the general population (Czlonkowska et al., 2018; Sanchez-Monteagudo et al., 2021). Due to the accumulation of copper ions in the body (liver, brain and cornea), typical symptoms of WD include liver function injury, extrapyramidal symptoms, mental symptoms and Kayser-Fleischer rings (K-F ring) (Collins et al., 2021; Shribman et al., 2021). Patients with WD usually present with a variety of clinical subtypes that are characterized by hepatic and/or nervous system manifestations. Due to its various clinical manifestations and the fact that it is one of the few treatable neurogenetic disorders, genetic testing is an important diagnostic tool for this disease (Espinos and Ferenci, 2020). The development of irreversible sequelae can be prevented by timely diagnosis and early interventions in patients with WD (Harada, 2014; Espinos and Ferenci, 2020).

In 1993, the gene that encodes a copper-transporter P-type ATPase (*ATP7B*) was defined as the causative gene of WD. It is composed of 1,465 amino acids that contain a phosphatase domain, a phosphorylation domain, a nucleotide-binding domain, six N-terminal metal binding domains and eight transmembrane ion channels (Telianidis et al., 2013). It is located on chromosome 13q14.3 and contains 21 exons and 20 introns (Tanzi et al., 1993). The protein is used to expel copper ions from the cell and it plays an important role in regulating copper ion metabolism (Tanzi et al, 1993). Worldwide, more than 1,000 different mutations have been identified in the *ATP7B* gene, most of which are missense, non-sense or frameshift mutations (Human Gene Mutation Database, HGMD, http://www.hgmd.cf.ac.uk/ac/index.php). When *ATP7B* mutations lead to the abnormal function of the P-type copper transporter ATPase, the ability of ceruloplasmin to bind and transport copper ions is decreased or removed, which results in the various clinical symptoms of WD (Chen et al., 2015; Ferenci et al., 2019).

To date, several studies have reported the spectrum and frequency of mutations in the *ATP7B* gene in the Chinese WD population. However, genetic profiling in different ethnic groups is still lacking, particularly in pediatric patients with WD. Due to the higher prevalence and more complicated correlation between genotype and phenotype in the Chinese population, when compared with Caucasians (Sanchez-Monteagudo et al., 2020; Couchonnal et al., 2021), it is necessary to study the *ATP7B* profiles of different ethnic groups in China. In this study of WD patients from Yunnan province, we conducted a mutation analysis in 39 patients and their family members and identified four novel mutations in the *ATP7B* gene.

## Materials and methods

### Patients and data collection

Forty-five patients with WD, from 44 families, were recruited between January 2017 and October 2022 at the Children’s Hospital Affiliated to Kunming Medical University. The age range of pediatric patients was 0–18 years. The medical history, physical examination results, laboratory test data and imaging results were collected as clinical data. Physical examinations included tests for jaundice, hepatomegaly, K-F rings and neurological symptoms. Laboratory tests included routine blood tests, biochemical liver tests, renal function tests, coagulation function tests, virological tests (hepatitis virus, cytomegalovirus and Epstein-Barr virus), ceruloplasmin and 24-hour urinary copper level. Imaging included liver ultrasound and brain magnetic resonance imaging (MRI). The patients were diagnosed in accordance with their clinical symptoms, biochemical parameters and/or genetic analysis (Czlonkowska et al., 2018). All patients were assessed using the Leipzig score and the total score was ≥4 in every patient. There were two groups used for classifying the patients: Group 1 represented the Han ethnicity and ethnic minorities, while Group 2 represented the general group (patients from general departments) and the severely affected group (patients from the pediatric intensive care unit). Direct sequencing of the *ATP7B* gene was performed in 39 of the 45 patients and their family members. Written informed consent was signed by custodians of the child participants. This study adhered to the Declaration of Helsinki and was approved by the Ethics Committee of the Children’s Hospital Affiliated to Kunming Medical University, Kunming, Yunnan, China (No.202303034K01).

### Light and electron microscopy analysis

Liver specimens from patient six were fixed in 10% formalin and embedded in paraffin. The thickness of the paraffin sections was 3 μm and they were stained with hematoxylin and eosin and Masson’s trichrome stain. The sections were observed under an Olympus BX53 microscope (Olympus, Tokyo, Japan). The sample for transmission electron microscopy was fixed in 2.5% glutaraldehyde for two hours, then fixed in 1% osmotic acid for one to two hours, dehydrated in acetone and embedded in epoxy resin. Ultrathin sections were sliced and stained with 3% uranyl acetate and lead citrate. The sections were observed under a transmission electron microscope (H7700, Hitachi, Tokyo, Japan).

### DNA extraction and genetic analysis

Peripheral blood was collected from each patient (5 mL) and their parents (2 mL). The blood samples were sent to Beijing Kangso Medical Inspection for *ATP7B* genetic testing. The Qiagen FlexiGene DNA kit was used to extract genomic DNA from blood samples. The primers were designed using the Primer Z website (http://genepipe.ncgm.sinica.edu.tw/primerz/primerz4.do). The mutation sites were amplified by PCR and then sequenced by first-generation sequencing. The PCR amplification conditions were: 10 min at 95°C; 35 cycles of 30 s at 95°C, 30 s at 60°C and 45 s at 72°C; followed by 5 min at 72°C. The PCR products were analyzed by electrophoresis on a 1% agarose gel and then sequenced using an ABI PRISM 3730XL DNA automated sequencer (Applied Biosystems; Thermo Fisher Scientific, Inc.).

The mutations identified in this study were compared to the list of reported pathogenic mutations in the Human Gene Mutation Database (https://www.hgmd.cf.ac.uk/ac/index.php), the Wilson Disease Mutation Database (http://www.wilsons-disease.org.uk/) and the gnomAD database (http://gnomad-sg.org/). The pathogenicity of the mutations was evaluated in strict accordance with the American College of Medical Genetics and Genomics (ACMG) Standards and Guidelines (Richards et al., 2015), using Sorting Intolerant From Tolerant (SIFT, http://sift-dna.org), PolyPhen-2 (http://genetics.bwh.harvard.edu/pph2/index.shtml) and Mutation Taster (https://www.mutationtaster.org/). Splice Ai (https://spliceailookup.broadinstitute.org/) was used to evaluate splice sites. The Alpha Fold 2 (Alpha Fold Protein Structure Database, https://alphafold.ebi.ac.uk/) and NetGene2-2.42 (https://mybiosoftware.com/netgene2-2-42-intron-splice-sites-human-c-elegans-a-thaliana-dna.html) sites were used for protein analysis.

### Statistical analysis

Statistical analyses were performed using SPSS version 23. Quantitative data were expressed as the mean ± SD or median and interquartile range (IQR). Categorical variables were given as absolute (number) and relative frequencies (%). To compare quantitative data, the Student’s t-test or Mann-Whitney *U* test was used for 2-group comparisons. The *X* ^*2*^ test or Fisher’s exact test was used to compare categorical variables, as appropriate. The criterion for a significant difference was a *p*-value of less than 0.05.

## Results

### Clinical features and laboratory data for the WD patients

Clinical data and laboratory findings for the WD patients are summarized in Table 1. In this study, we examined 45 pediatric patients with WD, of whom 17 (37.78%) were female and 28 (62.22%) were male. Their mean age of onset was 7.62 ± 3.46 years, with a range of 2.00–13.58 years. Simple elevated transaminases were found in 64.44% (29/45) of patients. The median ceruloplasmin level was 30 mg/L (range: 10–55 mg/L) and the median urine ketone in 24 h level was 171.2 ug (range: 99-445.65 ug). Ten patients, including one each from Bai, Dai, Zhuang, Yi, Hui and Jingpo, were from ethnic minorities, while the majority of patients were Han Chinese. Liver disease was found in 43 (95.56%) individuals and nine (20%) had brain disease. One patient (2.22%) presented with simple neurological symptoms. We found abnormalities in the basal ganglia, thalamus or brainstem in the craniocerebral MRI in seven out of the 45 (15.56%) patients. Eight (17.78%) patients presented with K-F rings. There was no significant difference in age of onset between Han patients and patients from ethnic minorities (*p >* 0.05). Male patients were more prevalent in our overall sample of pediatric patients, regardless of whether they were Han or members of an ethnic minority.

**TABLE 1:** Characteristics of 45 patients with Wilson’s disease clinical and laboratory data.

Patient characteristics	WD (*n* = 45)	Group 1			Group 2			
		Ethnic Han (*n* = 35)	Ethnicminorities (*n* = 10)	*p*-value		General (*n* = 38)	Severe (*n* = 7)	*p-value*
General Information								
Gender (Male/Female)	28/17	22/13	6/4	0.869	25/13		3/4	0.25
Age (years)	7.62 ± 3.46	7.60 ± 3.27	7.68 ± 4.28	0.441	7.08 ± 3.17		12.33 ± 2.09	0.004**
Clinical subtypes								
Hepatic	35 (77.78%)	27 (77.14%)	8 (80%)	0.893	32 (84.21%)		3 (42.86%)	0.031*
Neurologic	1 (2.22%)	1 (2.86%)	0		1 (2.63%)		0	
Mixed	8 (17.78%)	6 (11.14%)	2 (20%)		4 (10.53%)		4 (57.14%)	
Others	1 (2.22%)	1 (2.86%)	0		1 (2.63%)		0	
Affected organs								
Liver	43 (95.56%)	33 (94.29%)	10 (100%)	0.439	36 (94.74%)		7 (100%)	0.535
Brain	9 (20%)	7 (20%)	2 (20%)	1.000	5 (13.16%)		4 (57.14%)	0.008*
Eye (K-F ring)	8 (17.78%)	7 (20%)	1 (10%)	0.466	7 (18.42%)		1 (14.29%)	0.793
Liver function								
ALT (0–40IU/L)	136 (64–351.5)	139 (77.75–432.5)	82 (25–234)	0.098	148 (82–423)		30 (27–33)	0.002**
AST (0–40IU/L)	100 (70–231.5)	98 (71–285.75)	112 (61–182)	0.841	101.5 (74–281)		60 (50–104.5)	0.754
TB (1.71-17.1umol/L)	12.8 (8.7–27.3)	11.8 (8.7–18.95)	17.4 (10.4–69.5)	0.035*	11.8 (8.7–17.1)		343.6 (206.55–345.8)	0.000**
Serum total protein (60–80 g/L)	62.99 ± 6.65	63.27 ± 7.06	62.1 ± 5.53	0.206	63.82 ± 6.06		55.77 ± 8.58	0.013*
Albumin (35–50 g/L)	37.17 ± 6.42	37.81 ± 5.44	35.17 ± 9.1	0.140	38.41 ± 5.3		26.47 ± 5.93	0.000**
Copper related index								
Serum ceruloplasmin (200–400 mg/L)	30 (10–55)	20 (10–60.75)	30 (10–40)	0.324	20 (10–40)		60 (45–75)	0.094
Urine ketone in 24 h (15–60μg/24 h)	171.2 (99–445.65)	156.9 (92.13–441.63)	179.8 (133.19–987.6)	0.358	156.9 (95.9–296.39)		2,344.5 (2,326.5–3,172.25)	0.000**
Blood Coagulation Profile								
PLT (100–300×10^9/L)	258.31 ± 141.45	258.41 ± 149.3	258 ± 123.95	0.417	274.81 ± 138.88		115.33 ± 69.62	0.000**
PT (11–14.5s)	40.2 (31.75–46.4)	12.65 (12.05–15.02)	13.2 (11.4–24.5)	0.206	12.5 (11.9–14.4)		25.6 (24.7–27.35)	0.000**
APTT (28–44.5s)	40.16 ± 95.01	39.85 ± 9.21	41.16 ± 12.03	0.207	38.72 ± 9.18		52.17 ± 6.0	0.000**
FIB (2–4 g/L)	2.08 ± 0.60	2.2 ± 0.59	1.71 ± 0.52	0.203	2.16 ± 0.57		1.42 ± 0.47	0.000**
INR (0.8-1.5)	1 (0.94–1.42)	1 (0.95–1.18)	1.01 (0.92–2.14)	0.137	1 (0.93–1.04)		2.63 (2.39–2.70)	0.000**

Note: K-F, Kayser-Fleischer; ALT, alanine aminotransaminase; AST, aspartic transaminase; TB, total bilirubin; PT, prothrombin time; APTT, activated partial thromboplastin time; FIB, fibrinogen; INR, international normalized ratio; PLT, platelets. General: patients from general departments; Severe: patients from pediatric intensive care unit (PICU). *p < 0.05, **p < 0.01. “Others” indicates that the function of organs other than the liver and brain is impaired.

The laboratory data showed that, when compared to Han patients, ethnic minority patients had higher total bilirubin (TB) (*p* = 0.035). We also found that severely affected patients were significantly older at the onset of symptoms than general patients (*p* = 0.004). The severely affected patient group had a higher percentage of individuals with mixed subtype (*p* = 0.031) and symptoms that affected the brain (*p* = 0.008). General patients had increased alanine aminotransaminase (ALT), while severely affected patients had a normal ALT range (*p* = 0.002). Increased TB, prothrombin time (PT), activated partial thromboplastin (APTT), International Normalized Ratio (INR), urine ketone in 24 h and decreased platelets (PLT), serum total protein, albumin and fibrinogen (FIB) were observed in severely affected patients (*p* < 0.05).

Three pediatric patients underwent a liver biopsy during hospitalization. As shown inFigure 1, hepatocyte steatosis, cloudy swelling and ballooning were commonly observed. Glycogenated nuclei were present in hepatocytes and the Masson’s trichrome stain showed hepatic fibrotic lesions. Ultrastructural analysis showed pleomorphic dilated mitochondria.

**FIGURE 1:**
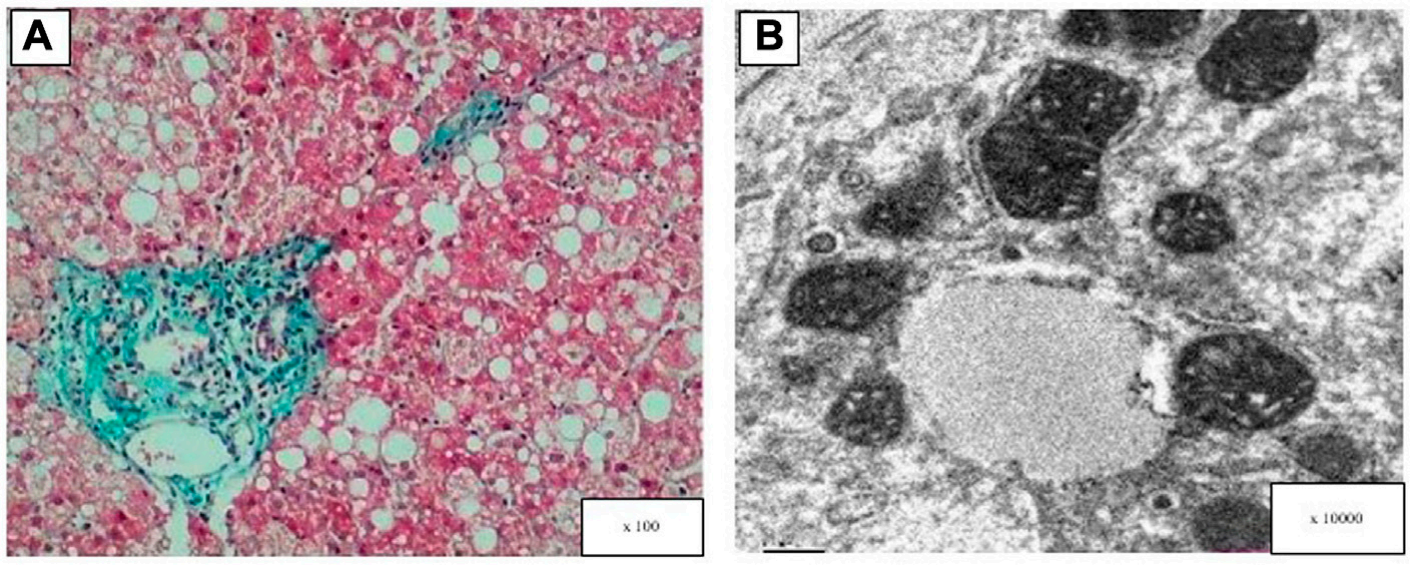
Light and electron microscopy analysis of the patient’s liver (patient 6). **(A)** Masson’s trichrome stain showed hepatic fibrotic lesions, diffuse hepatocellular steatosis (the size of the bubble fat gets mixed, 70%), and a few hepatocytes showed balloon-like. **(B)** Electron microscopy: hepatocyte swelling and variable lipid droplets are seen in diffuse hepatocytes.

### Characterization of genetic mutations in the *ATP7B* gene

**FIGURE 2:**
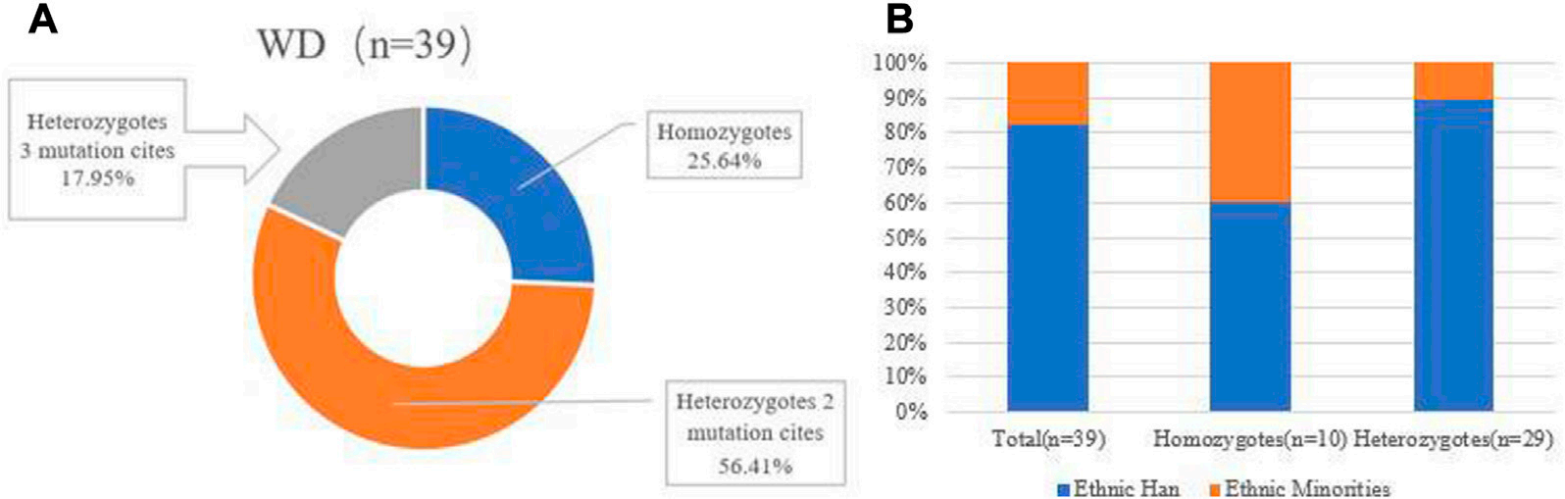
**(A)** Distribution of genotype in total patients with WD. **(B)** Distribution of genotype in patients from different ethnic groups (*p* = 0.035).

A total of 39 pediatric patients, from 38 unrelated families, were analyzed by direct sequencing. The results are shown in Table 2. Patient 20 and patient 21 were from the same family. Among the 39 patients, 25.64% (10/39) harbored homozygous mutations and 74.36% (29/39) harbored compound heterozygous mutations. Of the patients that harbored compound heterozygous mutations, 75.86% (22/29) had two mutations and 24.14% (7/29) had three mutations (Figure 2A). Interestingly, we observed that the c.2310C > G mutation always accompanied the c.2333G > T mutation (100%), while the c.2333G > T mutation was either a homozygous site or was found with other mutations. In the patients that had three mutations, it was noted that 71.43% (5/7) had the combination of c.2310C > G and c.2333G > T. Phenotype-genotype correlation analysis suggested that, when compared with Han patients, patients from ethnic minorities tended to have homozygous mutations (*p* = 0.035) (Figure 2B).

**TABLE 2:** Clinical and genetic data of the 39 patients with WD.

Patient	Gender	Ethnic group	Age at presentation(y)	ATP7B genotypes				
				Patient			Father	Mother
1	Female	Han	4.5	c.1708–5T > G			c.1708–5T > G	c.1708–5T > G
2	Female	Han	3.83	c.2975C > T/c.1286-1delG			c.2975C > T	**c.1286-1delG**
3	Male	Han	3	c.1708-1G > C/c.1543+1G > T			c.1708-1G > C	c.1543+1G > T
4	Male	Han	5.5	c.2333G > T/c.3443T > C			c.3443T > C	c.2333G > T
5	Male	Han	12.92	c.2549C > T/c.3809A > G			c.3809A > G	c.2549C > T
6	Male	Han	3.17	c.2333G > T/c.2297C > T/c.2310C > G			c.2333G > T/c.2310C > G	c.2297C > T
7	Male	Han	8.92	c.2621C > T/c.3859G > A			c.3859G > A	c.2621C > T
8	Male	Han	4.5	c.2310C > G/c.2333G > T/c.2755C > G			c.2755C > G	c.2310C > G/c.2333G > T
9	Female	Han	6.58	c.3443T > C/c.2310C > G/c.2333G > T			c.3443T > C	c.2310C > G/c.2333G > T
10	Male	Han	7.58	c.2333G > T/c.4112T > C			c.2333G > T	c.4112T > C
11	Female	Han	7.25	c.2120A > G			—	c.2120A > G
12	Male	Han	8.58	c.2333G > A/c.2668G > A			c.2668G > A	c.2333G > A
13	Male	Han	7.75	c.2212_2213delAGinsCA/c.2333G > T			—	—
14	Male	Han	4	c.2294A > G/c.3089G > A			—	—
15	Female	Han	13.58	c.3809A > G/c.3818C > A			—	—
16	Female	Han	9	c.2975C > T/c.2333G > T			—	c.2333G > T
17	Male	Han	3	c.2621C > T/c.2570_2572del			c.2621C > T	**c.2570_2572del**
18	Male	Han	4	c.2668G > A/c.2333G > T/c.2310C > G			c.2333G > T/c.2310C > G	c.2668G > A
19	Male	Han	4.5	c.2621C > T			c.2621C > T	c.2621C > T
20	Male	Han	10.5	c.3993T > G/c.2804C > T			c.3993T > G	c.2804C > T
21	Female	Han	5.58	c.3993T > G/c.2804C > T			c.3993T > G	c.2804C > T
22	Male	Han	5	c.2333G > T/c.2310C > G			c.2310C > G	c.2333G > T
23	Male	Han	11.42	c.2333G > T			c.2333G > T	c.2333G > T
24	Female	Han	5.92	c.2662A > C			c.2662A > C	c.2662A > C
25	Female	Han	6.17	c.2975C > T/c.2332C > T			c.2975C > T	c.2332C > T
26	Male	Han	2	c.3742A > G/c.2304dupC			c.2304dupC	**c.3742A** > **G**
27	Male	Han	9.92	c.2145C > A/c.2299C > T			c.2299C > T	c.2145C > A
28	Female	Han	9.92	c.2621C > T			not found	c.2621C > T
29	Male	Han	9	c.1870–8A > G/c.2804C > T			c.2804C > T	c.1870–8A > G
30	Male	Han	3	c.3532A > G/c.1869 + 20A > G/c.2121+3A > G			**c.1869 + 20A** > **G**	c.3532A > G
							/c.2121+3A > G	
31	Female	Han	8.25	c.2333G > T/c.1369C > T/c.2310C > G			c.2333G > T/c.2310C > G	c.1369C > T
32	Male	Han	3.33	c.2975C > T/c.2662A > C			c.2662A > C	c.2975C > T
33	Male	Bai	3	c.2785A > G/c.2906G > A/c.3809A > G			—	c.2785A > G/c.2906 G > A
34	Male	Zhuang	10.17	c.3079G > C			c.3079G > C	c.3079G > C
35	Female	Yi	10.58	c.2228A > G/c.3932T > A			c.3932T > A	c.2228A > G
36	Male	Dai	13.5	c.3079G > C			c.3079G > C	c.3079G > C
37	Female	Dai	7.33	c.4112T > C			c.4112T > C	c.4112T > C
38	Male	Bai	3.42	c.3809A > G/c.2333G > T			c.2333G > T	c.3809A > G
39	Male	Jingpo	12	c.3932T > A			c.3932T > A	—

Note: Novel mutations are highlighted in bold. Gene reference sequence: GRch38, NM_000053.4; Protein reference sequence:NP_000044.2.

**FIGURE 3:**
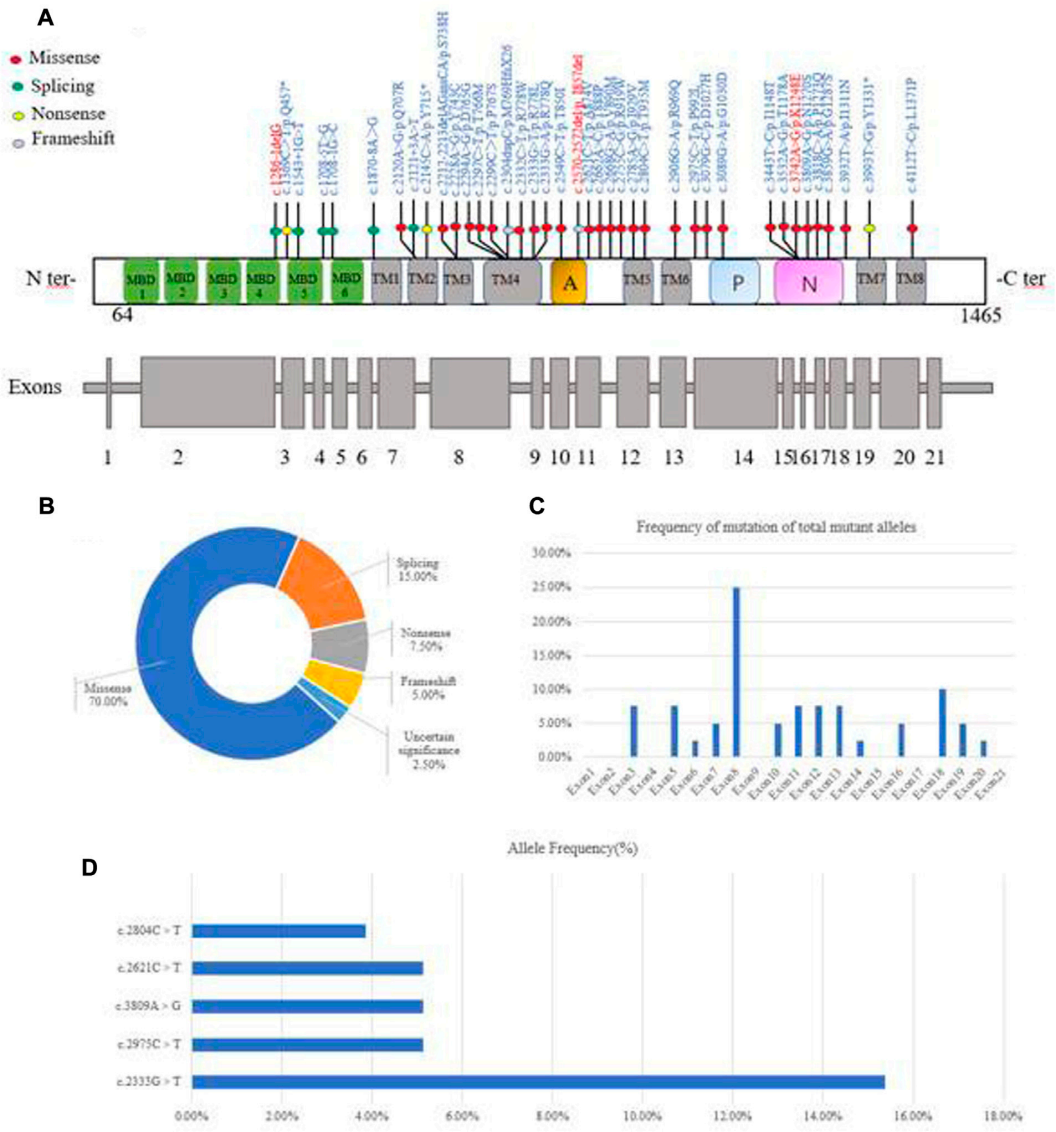
Characterization of genetic mutations in the *ATP7B* gene. **(A)** Novel mutations were observed. **(B)** Various mutants identified in this study and their percentage. **(C)** The frequency of mutations found in the 39 WD patients is given per exon as a percentage of the total mutant alleles. **(D)** Allele frequency of common *ATP7B* mutations in Yunnan province. MBDs, metal binding domains; Transmembrane segments are numbered TM1 to TM8; A: actuator domain; *p*: phosphorylation domain; N: nucleotide-binding domain.

In 39 patients with WD, 39 potential pathogenic *ATP7B* mutations, one known polymorphism (c.2310C > G) that does not disrupt gene function and one mutation of uncertain significance (c.1869 + 20A > G) were identified (Figure 3A). Four of the mutations were novel (c.1286-1delG, c.1869 + 20A > G, c.2570_2572del and c.3742A > G). The mutations consisted of 28 (70%) missense mutations, six (15%) splice-site mutations, three (7.5%) non-sense mutations, two (5%) frameshift mutations and one (2.5%) mutation of uncertain significance (Figure 3B). The mutations were distributed in all exons apart from exons 1, 2, 4, 9, 15, 17 and 21. We found that exon 8 (26.83%) had the highest frequency of mutations, followed by exon 18 (9.76%), exon 3 (7.32%), exon 11 (7.32%), exon 12 (7.32%) and exon 13 (7.32%). This suggested that these exons may be more susceptible to mutations that cause WD in Yunnan province (Figure 3C). The most frequent mutation was c.2333G > T (p.R778L, allelic frequency: 15.38%), followed by c.2975C > T (p.P992L, 5.26%), c.2621C > T (p.A874V, 5.13%), c.3809A > G (p.N1270S, 5.13%) and c.2804C > T (p.T935M, 3.85%) (Figure 3D). The allelic frequency of c.2333G > T and c.2975C > T showed the same trend, in terms of the Minor allele frequency in genomAD from East Asian (Minor allele frequency in genomAD from East Asian). The allelic frequency of c.2621C > T and c.3809A > G was 5.13% (4/78), while the MAF was 0.0000. We also found that the allelic frequency c.2785A > G was 1.28% (1/78), which was not consistent with the MAF (0.0166) (Table 3).

**TABLE 3:** The potential pathogenic *ATP7B* mutations.

Number	Loci	Nucleotide	rs ID	MAF	Allelic count
1	Exon3	**c.1286-1delG**	chr13:52544885-52544886delG	—	1/78
2	Exon3	c.1369C > T	rs1951795080	0.0000	1/78
3	Exon3	c.1543+1G > T	rs1360279134	0.00002	1/78
4	Exon5	c.1708–5T > G	rs770829226	0.0003	1/78
5	Exon5	c.1708-1G > C	rs137853280	0.0003	1/78
6	Exon5	**c.1869 + 20A > G**	chr13:52538988A>G	—	1/78
7	Exon6	c.1870–8A > G	chr13:52536057A>G	—	1/78
8	Exon7	c.2120A > G	chr13:52534285A>G	—	1/78
9	Exon7	c.2121+3A > T	rs1248002612	0.0000	1/78
10	Exon8	c.2145C > A	rs751202110	0.0000	1/78
11	Exon8	c.2212_2213delAGinsCA	chr13:52534293delAGinsCA	—	1/78
12	Exon8	c.2228A > G	chr13:52532574A>G	—	1/78
13	Exon8	c.2294A > G	chr13:52523826A>G	—	1/78
14	Exon8	c.2297C > T	rs121907997	0.0006	1/78
15	Exon8	c.2299C > T	rs2139531503	—	1/78
16	Exon8	c.2304dupC	chr13:52532499dupC	—	1/78
17	Exon8	c.2332C > T	rs137853284	0.0000	1/78
18	Exon8	c.2333G > T/A	rs28942074	0.0019	13/78
19	Exon10	c.2549C > T	rs777629392	0.0003	1/78
20	Exon10	**c.2570_2572del**	chr13:52524411–52524413 del TCA	—	1/78
21	Exon11	c.2621C > T	rs121907994	0.0000	4/78
22	Exon11	c.2662A > C	rs1455758826	0.0000	2/78
23	Exon11	c.2668G > A	rs786204718	0.0000	1/78
24	Exon12	c.2755C > G	rs121907993	0.0000	1/78
25	Exon12	c.2785A > G	rs534960245	0.0166	1/78
26	Exon12	c.2804C > T	rs750019452	0.0003	3/78
27	Exon13	c.2906G > A	rs121907996	0.0000	1/78
28	Exon13	c.2975C > T	rs201038679	0.0006	4/78
29	Exon13	c.3079G > C	rs1593672840	—	2/78
30	Exon14	c.3089G > A	chr13:52520548G>A	—	1/78
31	Exon16	c.3443T > C	rs60431989	0.0000	2/78
32	Exon16	c.3532A > G	rs1387431334	0.0000	1/78
33	Exon18	**c.3742A** > **G**	chr13:52513301A>G	—	1/78
34	Exon18	c.3809A > G	rs121907990	0.0000	4/78
35	Exon18	c.3818C > A	rs758355520	0.0000	1/78
36	Exon18	c.3859G > A	rs762866453	0.0000	1/78
37	Exon19	c.3932T > A	chr13:52511501T>A	—	2/78
38	Exon19	c.3993T > G	chr13:52511440T>G	—	2/78
39	Exon20	c.4112T > C	rs1444841250	0.0000	2/78

Note: MAF, Minor Allele frequency in genomAD from East Asian. Novel mutations are highlighted in bold.

### Characterization of novel mutations

**FIGURE 4:**
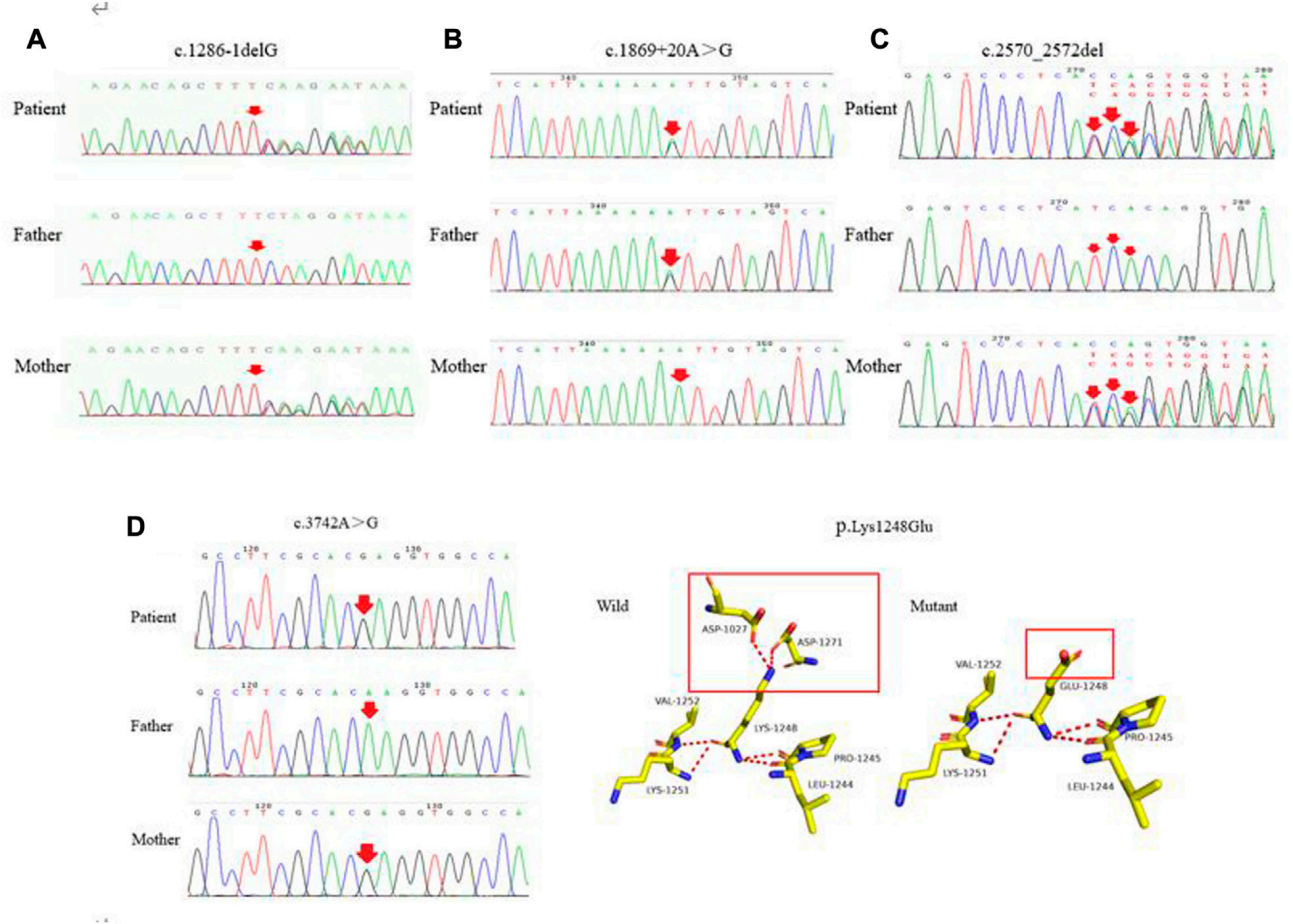
**(A–C)** represent the novel mutations (c.1286-1delG, c.1869 + 20A > G, c.2570_2572del), respectively. **(D)** c.3742A > G was detected in the patient and his mother. As shown in the structure of *ATP7B* protein, each color represents different atoms, yellow - C atom, gray - H atoms, blue - N atoms, red - O atoms, orange - S atom, the red dotted line for the hydrogen bond. The red rectangles represent areas of change.

The four novel mutations found in the pediatric patients with WD included one splice-site mutation, one missense mutation, one frameshift mutation and one mutation of uncertain significance. As shown in Figure 4A, the novel c.1286-1delG mutation was identified in the *ATP7B* gene of a patient and her mother. Using Splice Ai, we predicted the impact of the mutation on the protein. The NetGene2-2.42 site was used to predict that c.1286-1delG would cause a shift in the splice-site. We also identified the novel c.1869 + 20A > G mutation in a patient and his mother, as shown in Figure 4B. Splice Ai predicted that this mutation would not impact the splice-site. As shown in Figure 4C, the novel c.2570_2572del mutation was detected in a patient and his mother. Figure 4D shows the c.3742A > G mutation that was detected in another patient and his mother. The SIFT, PolyPhen-2 and Mutation Taster programs were used to predict the functional damage to the protein, caused by the mutations. The mutations shown in Figures 4B–D were classed as “Deleterious,” “Probably damaging” and “disease causing,” respectively. The structural analysis showed that the c.3742A > G (p.K1248E) mutation leads to the loss of the hydrogen bond between amino acids 1,027 and 1,271, which may affect the protein configuration (Figure 4D)

In conclusion, in accordance with the ACMG Standards and Guidelines, c.1286-1delG was considered to be a “pathogenic variant,” c.2570_2572del and c.3742A > G were considered to be “likely pathogenic variants,” while c.1869 + 20A > G was classified as a “variant with uncertain significance.” Further studies are needed to confirm the changes predicted by the *in silico* tools.

### Correlation between genotype and phenotype

To describe the correlation between the genotype and phenotype, we first studied the association between the exons and clinical subtypes in Yunnan province. The exons that were hotspots were examined in the 39 WD patients. The results showed that exons 8 and 18 harbored the highest percentage of mutations, which was not consistent with previous results that exons 8, 13 and 16 were the hotpot exons in the Chinese population (Li et al., 2021). This result may be due to the differences between Han individuals and ethnic minorities.

**FIGURE 5:**
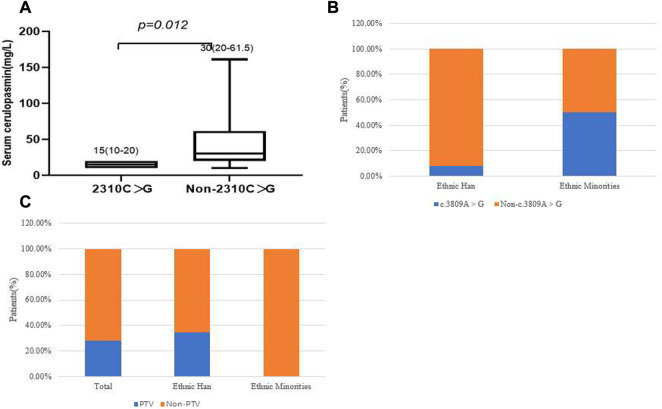
**(A)** Correlation of c.2310C > G and serum ceruloplasmin level. **(B)** correlation of c.3809A > G and ethnic groups. **(C)** The frequency of PTV in different ethnic groups.

We then focused on the correlation between specific mutations and phenotypes. The most prevalent mutations were examined in the 39 WD patients. There was no significant difference, in terms of age of onset, between different clinical subtypes and several common mutations (c.2333G > T, c.2975C > T, c.2621C > T and c.3809A > G), in the different ethnic groups (*p >* 0.05). The results showed that the two most frequent variants were c.2333G > T (p.R778L) and c.2975C > T (p.P992L). We observed that the patients who carried the c.2310C > G mutation had lower serum ceruloplasmin levels than patients with other mutations (*p* = 0.012, Figure 5A). In patients who harbored compound heterozygous mutations, those from ethnic minorities tended to carry c.3809A > G (*p* = 0.042, Figure 5B). We also analyzed the relationship between the phenotypes and PTV (protein-truncated variants, e.g., frameshift, non-sense and splice-sites). The frequency of PTV in patients from Yunnan province was 28.2% (11/39). Interestingly, we found that the frequency of PTV in Han patients was 34.38% (11/32), while we did not find any PTV in patients from ethnic minorities (Figure 5C). Among the patients with PTV, 90.91% (10/11) presented with elevated transaminases and the p.T935M/PTV genotype had a frequency of 27.27% (3/11).

## Discussion

To define the mutation spectrum, clinical characteristics and genotype-phenotype correlations in WD in various ethnic groups, we examined *ATP7B* mutations in 39 pediatric patients from Yunnan province. Patients from seven ethnic groups (Han, Bai, Dai, Zhuang, Yi, Hui and Jingpo) from China were involved in this study. Patients with neurologic symptoms were not predominant in our cohort, in contrast to previous studies in China (Dong et al., 2021; Zhang et al., 2022). This may be because patients with neurologic symptoms mainly present during adolescence. However, male pediatric patients were predominant in our study, which is consistent with some studies in China and Korea (Cheng et al., 2017; Seo et al., 2018; Zhang et al., 2022). Most Han patients presented with elevated transaminases, while only three out of 10 patients from ethnic minorities presented with elevated transaminases. Three out of the six severely affected patients were from ethnic minorities. Several studies have shown that clinically asymptomatic patients have an earlier age of onset than patients with typical clinical manifestations (Li et al., 2021; Zhang et al., 2022). In our study, most pediatric patients presented with abnormal liver enzymes, without symptoms. We also found that pediatric patients from ethnic minorities had more severe disease.

This study identified homozygous mutations in 10 pedigrees and compound heterozygous mutations in 29 pedigrees. The frequency of homozygous mutations was 25.64% (10/39), which is not consistent with the frequency of 15.67% (204/1,302) that was observed in a previous study (Zhang et al., 2022). Moreover, phenotype-genotype correlation analysis suggested that, when compared with Han patients, patients from ethnic minorities tend to have homozygous mutations. This may indicate the significant genetic characteristics of patients from ethnic minorities from China. Of the patients who had compound heterozygous mutations, seven had three mutations. We observed that the c.2310C > G mutation always accompanied the c.2333G > T mutation, which is consistent with a previous study (aL). The c.2333G > T mutation was found as a homozygous site and also accompanied other mutations. It is possible that the coexistence of c.2333G > T and c.2310C > G may affect the function of the protein in a specific way. Hence, it is necessary to further explore the functional implications of both mutations.

Forty potential pathogenic mutations were identified in this study. Thirty-six of these have already been reported as disease-causing mutations in the Wilson Disease Mutation Database. The other four mutations were novel and evidence indicated that c.1286-1delG is considered to be a “pathogenic variant,” c.2570_2572del and c.3742A > G are considered to be “likely pathogenic variants,” while c.1869 + 20A > G needs further functional analysis and is classified as a “variant with uncertain significance.” In the patients who carried three mutations, it was noted that 71.43% had the c.2310C > G mutation, whilst the accompanying two pathogenic mutations had previously been reported. The novel c.1869 + 20A > G mutation co-occurred with two other reported pathogenic mutations (c.3532A > G and c.2121+3A > G) and its pathogenicity should be explored further.

The top five most common mutations in our study were c.2333G > T (p.R778L), c.2975C > T (p.P992L), c.2621C > T (p.A874V), c.3809A > G (p.N1270S) and c.2804C > T (p.T935M), which is not consistent with other reported studies (p.R778L, p.P992L, p.A874V, p.R919G and p.V1216M) (Li et al., 2021). Mutation hotspots in *ATP7B* vary by geographic region, with a higher prevalence of specific mutations reported in certain populations (Wallace and Dooley, 2020). The predominant mutations in the Chinese population include c.2333G > T (p.R778L), c.2975C > T (p.P992L), c.3443T > C (p.I1148T) and c.2804C > T (p.T935M). In contrast, the most common mutation in European populations is p.H1069Q (Wang et al., 2011; Wei et al., 2014). Chinese populations from different regions also have different mutation types. Although p.R778L was always detected as the most common pathogenic mutation, a study of populations in Northern China found that p.A874V was the second most common pathogenic mutation, whilst p.I1148T was the second most common mutation found in Guangdong province, Southern China (Li et al., 2013; Wei et al., 2014). Another study found that in pediatric patients from Southern China, p.R778L and p.I1148T had the highest frequencies, at approximately 23.0% and 10.7%, respectively. This is not consistent with our study (Zhou et al., 2022). A previous study found that p.T935M was significantly associated with Fujian province, which hints at the possibility of a founder effect (Wei et al., 2014). In our study, p.T935M was found in Yunnan province. The gnomAD database also shows a low frequency of p.T935M in the East Asian population, which does not support the previous hypothesis of a founder effect. Due to the vast diversity of mutations in the *ATP7B* gene, analysis of the regional distribution of mutations can help to develop time-saving approaches and speed up the genetic diagnosis of WD in specific regions (Roy et al., 2020).

The overall genetic diagnosis rate in this study was 97.5% (39/40). Previous studies gave 78.4% in Caucasians, by exon-by-exon sequencing, and 87.9% in Poland, by whole-exome sequencing (Ferenci et al., 2019; Kluska et al., 2019). In China, one study showed that the genetic diagnosis rate was 90.0%, by sequencing of the 5’ untranslated region (UTR), 21 exons and their flanking regions (Dong et al., 2016). Another study estimated that the genetic diagnosis rate was 97.1%, by mutational analysis of 68 WD patients from China (aL). The reason that not all patients are genetically diagnosed may be related to a large hemizygous deletion, regulatory region variants and genetic alterations outside of the *ATP7B* gene (Dong et al., 2016; Ferenci et al., 2019).

We did not find a significant difference in terms of age of onset between different clinical subtypes and several common mutations (c.2333G > T, c.2975C > T, c.2621C > T and c.3809A > G) in different ethnic groups (*p* > 0.05). This may have been due to the small sample size. Therefore, the sample size in Yunnan province should be expanded further to study these correlations.

We also analyzed the relationship between phenotypes and PTV. The frequency of PTV in patients from Yunnan province was 28.2% (11/39), which is consistent with the previous finding that the frequency of PTV was 26.5% (345/1,302) in a large cohort of patients with WD (Zhang et al., 2022). Interestingly, we found that the frequency of PTV in Han patients was 34.38% (11/32), while we did not find PTV in patients from ethnic minorities. Among the patients with PTV, 90.91% (10/11) presented with elevated transaminases and a specific genotype (p.T935M/PTV) had a frequency of 27.27% (3/11). Previous studies have found that truncating variants are associated with an early onset of WD (Gromadzka et al., 2005; Merle et al., 2010). Recently, one study found that PTV was more common in patients with a younger age of onset in both the hepatic and neurological groups (Zhang et al., 2022). There has been no research into the role of PTV in different ethnic groups. However, our findings revealed that PTV are not common in ethnic minority patients.

## Conclusion

In this study, we performed mutation analysis of the *ATP7B* gene in 39 WD patients from Yunnan province. In summary, our study identified four novel mutations that expand the spectrum of pathogenic *ATP7B* mutations. In addition, phenotype-genotype correlation analysis suggested that, when compared with Han patients, patients from ethnic minorities tend to carry homozygous mutations. We observed that the patients who carried the c.2310C > G mutation had lower serum ceruloplasmin levels than patients with other mutations. In patients with heterozygous mutations, the c.3809A > G mutation was significantly associated with ethnic minorities. The frequency of PTV in patients from ethnic minorities was lower than in Han patients. Our research highlights the differences in genotypes of WD pediatric patients from different ethnic groups. This may provide valuable insights into the diagnosis, counseling and treatment of WD pediatric patients in China.

## Data Availability

The original contributions presented in the study are included in the article/supplementary materials, further inquiries can be directed to the corresponding authors.
